# Pre-analytical characterization of CNS-derived extracellular vesicles from human saliva: effect of room temperature and cellular origin

**DOI:** 10.3389/fnins.2026.1765229

**Published:** 2026-03-23

**Authors:** Luciano M. Licatini, Luigi M. Licatini, Fadi A. Haddadin, Grace C. Conklin, AmanPreet Badhwar, Floyd M. Sarsoza, Sonal Sukreet, Charisse N. Winston

**Affiliations:** 1 Alzheimer’s Therapeutic Research Institute - Neuroscience Translational Research Division, Department of Physiology and Neuroscience, Keck School of Medicine of the University of Southern California, San Diego, CA, United States; 2 University of California San Diego, San Diego, La Jolla, CA, United States; 3 San Diego State University, San Diego, CA, United States; 4 Multiomics Investigation of Neurodegenerative Diseases (MIND) Lab, Centre de Recherche de l’Institut Universitaire de Gériatrie de Montréal (CRIUGM), Montreal, QC, Canada; 5 Department of Pharmacology and Physiology, Faculty of Medicine, University of Montreal, Montreal, QC, Canada; 6 Institute of Biomedical Engineering, University of Montreal, Montreal, QC, Canada

**Keywords:** Alzheimer’s disease, biomarkers, extracellular vesicles, pre-analytical measures, saliva, storage temperature

## Abstract

**Introduction:**

Blood-derived extracellular vesicles (EVs) from neurons and astrocytes carrying Alzheimer’s disease (AD) biomarkers can predict progression from mild cognitive impairment (MCI) to AD; however, their potential in saliva remains largely unexplored. Saliva-derived extracellular vesicles (sEVs) represent a promising non-invasive biomarker source for AD and other age-related dementias (ADRD), but progress has been limited by a lack of standardized protocols for saliva collection, storage, and central nervous system (CNS)-derived EV isolation.

**Methods:**

This study had two primary objectives: (1) to optimize enrichment of CNS cell-specific sEVs from the same individuals, and (2) to evaluate the impact of cellular origin and storage temperature (room temperature, 4°C, −20°C) on the stability and quantification of AD-related biomarkers and inflammatory cytokines. Saliva was collected via passive drool from participants in the Nathan Shock Healthy Aging Study (mean age 71.3 years; n = 15). EVs of neuronal, astrocytic, microglial, and oligodendrocyte origin were isolated using ExoQuick-TC precipitation followed by magnetic bead immunocapture. Executive function and attention were assessed using the NIH Toolbox Cognition Battery. Biomarkers were quantified using high-sensitivity immunoassays (MSD, SIMOA Qunaterix).

**Results:**

Astrocyte-derived EVs demonstrated significant enrichment of key AD biomarkers, including Aβ40, Aβ42, and total tau. Phosphorylated tau (p-tau217) was largely undetectable across all fractions. TDP-43 was most abundant in EV-depleted saliva, while inflammatory cytokines were broadly distributed across all fractions. Storage temperature did not consistently alter biomarker levels; however, −20°C storage yielded optimal biomarker quantification. Importantly, lower levels of inflammatory cytokines (IFN-γ, IL-10, and IL-6) in EV-depleted saliva were associated with better working memory performance.

**Discussion:**

This study provides proof-of-concept validation for the characterization and comparison of multiple CNS-derived salivary EV fractions within the same individuals. The findings support saliva as a feasible, non-invasive matrix for assessing neurodegenerative and neuroinflammatory biomarkers. Establishing a standardized methodology for salivary EV isolation and storage lays the groundwork for future longitudinal studies aimed at diagnosing and predicting AD progression using saliva-based biomarkers.

## 1 Introduction

Alzheimer’s disease (AD) is a progressive neurodegenerative disease characterized by cognitive decline and neuronal loss. This global health epidemic has affected over 50 million people globally and is projected to quadruple in size by 2050 (Breijyeh and Karaman, 2020). The lack of effective treatment and curative therapies makes resolving the AD epidemic increasingly urgent.

Early diagnostics and treatments are important, as interventions are most effective when implemented during preclinical stages (Rasmussen and Langerman, 2019). The current state of early diagnostics for AD includes cerebrospinal fluid (CSF) testing and neuroimaging techniques, specifically, positron emission tomography (PET; Juganavar et al., 2023). These methods measure biomarkers associated with the ATN framework, which designates three biomarker categories as central to AD pathology: Aβ deposition (A), pathologic tau (T), and neurodegeneration (N; Jack et al., 2024; Dubois et al., 2024). This framework provides a standardized method for classification based on biomarker quantification, supports the early detection of disease development, and streamlines research focused on developing disease therapies. However, despite their diagnostic value, these methods are often costly, require specialized personnel and repeated clinical visits, and can be invasive for patients (Lee et al., 2017). These limitations have shifted research efforts toward testing for levels of ATN biomarkers in blood. Although blood collection is less invasive than CSF testing and more accessible than neuroimaging, it comes with its own set of limitations. Blood collection is still invasive, as it requires a needle stick that causes pain or discomfort, and demands specialized staff for collection and processing, limiting its accessibility in low-income and rural communities. These limitations also include difficulties related to at-home testing, transportation, and stability of the sample, and often require multiple visits for long-term monitoring (Kim et al., 2022). More critically, most biomarker validations in blood have focused on non-Hispanic White (NHW) cohorts, despite non-Hispanic Black (NHB) and Hispanic individuals being approximately twice as likely to develop AD. This severe health equity deficit means the biomarker utility of blood-based biomarkers remains unknown for the populations most at risk.

Pathological hallmarks of AD, including the abnormal accumulation of Aβ plaques in the brains of individuals with AD, has also been found in several peripheral biofluids besides blood, including nasal mucosa, urine, saliva, and tears (Breijyeh and Karaman, 2020). Among the peripheral biofluid tissues mentioned, saliva collection stands out due its non-invasive nature, requires minimal training or specialized staff, and potentially avoids complex pre-analytical collection and processing challenges inherent to other biofluids like blood and CSF (Cui et al., 2024). Building on this peripheral presence, recent studies detecting phosphorylated tau (p-tau) and interleukin-34 (IL-34) in saliva suggest they can serve as promising biomarkers for both preclinical and clinical stages of AD, further underscoring saliva’s potential as a noninvasive diagnostic medium (Bamford et al., 2025; Boschi et al., 2022; Sabbagh et al., 2018; Clark et al., 2012).

Pioneering work identifying salivary amyloid as a potential AD biomarker reported elevated Aβ levels in mild AD compared to healthy controls, a distinction not observed in patients with Parkinson’s disease (Bermejo-Pareja et al., 2010). While salivary biomarkers have recently been successfully employed for conditions like COVID-19 (Weber et al., 2024) and sleep apnea (Bencharit et al., 2021) the medium poses reproducibility challenges. Potential contamination by bacteria and food particles necessitates rigorous and standardized collections protocols that account for oral health and diet (Sjoqvist and Otake, 2023). Moreover, the systemic origin of these proteins introduces peripheral confounding that can mask CNS-specific pathology (Hansson, 2021). These limitations have prompted a shift toward extracellular vesicle (EV) research for AD biomarker discovery.

EVs are nano-sized, membrane-bound vesicles (40-100 nm) that mediate intercellular communication under both physiological and pathological conditions (Doyle and Wang, 2019). EVs are released by various cell types, including neurons, astrocytes, and oligodendrocytes, and carry diverse contents, which include proteins, lipids, and miRNAs (Doyle and Wang, 2019). These are thought to be reflective of the intracellular environment of the parent cell (Doyle and Wang, 2019). EVs are secreted by cells into bodily fluids such as blood, CSF, saliva, urine, and tears and have emerged as valuable reservoirs of biomarkers for neurodegenerative diseases (Doyle and Wang, 2019; Sun and Chang, 2024).

Initial EV-based research for early AD diagnosis has focused on plasma EVs (Boyer et al., 2024; Tian et al., 2023; Winston et al., 2016; Winston et al., 2019a). Studies have shown that plasma EVs carry ATN biomarkers which are found in altered levels in patients with AD and related dementias (ADRD; Lazar et al., 2022). This has led the field to recognize that changes in expression levels of Aβ, p-tau species, and complement proteins from CNS-derived EVs in plasma can accurately predict conversion of mild cognitive impairment (MCI) to AD (Winston et al., 2016). Many studies have demonstrated that specifically CNS-derived EVs in plasma offer a significant diagnostic advantage over total EVs, which primarily reflects general systemic changes in the periphery (Winston et al., 2016; Lee et al., 2021; Winston et al., 2022; Winston et al., 2019a; Upadhya et al., 2020; Badhwar et al., 2024; Winston et al., 2019b).

The demonstrated potential of blood EV analysis, coupled with the logistical limitations of blood-based testing, has driven growing interest in saliva EVs (sEVs) as a more accessible source for AD biomarker discovery (Reseco et al., 2024; Cui et al., 2024; Sjoqvist and Otake, 2023; Sun and Chang, 2024; Lazar et al., 2022; Weber et al., 2024). EVs originating in the brain cross the blood–brain barrier (BBB) and, due to the connection between salivary glands and systemic circulation, eventually infiltrate the salivary environment (Cui et al., 2024). Crucially, concentrations of ATN biomarkers in saliva EVs (sEVs) may be altered prior to symptom onset, offering a window for the early diagnosis of neurodegenerative diseases. Consequently, characterizing the molecular cargo of sEVs represents an untapped, high-potential avenue for biomarker discovery.

The detection of sEVs has been hindered by unstandardized protocols for collection, storage, and the specific isolation of CNS-derived vesicles. While previous studies have investigated various isolation techniques (Reseco et al., 2024; Cui et al., 2024), optimal workflows remain undefined. This study aims to evaluate the feasibility of sEVs as biomarkers for potential early AD detection through two primary objectives. First, we compared biomarker concentrations across CNS-derived sEVs and EV-depleted saliva to determine how EV enrichment influences biomarker detection. Second, we assessed the impact of storage temperature on biomarker stability in CNS-derived sEVs. Since optimal storage is critical for preventing cargo leakage and bilayer damage (Sivanantham and Jin, 2022), we analyzed samples stored at room temperature, 4 °C, and −20 °C to identify the conditions that best preserved biofluid utility.

## 2 Materials and methods

### 2.1 Patient demographics and saliva collection

Saliva samples were acquired through the Nathan Shock Healthy Aging Study at the University of California, San Diego (UCSD; PI Molina, Protocol #201141; Phang et al., 2024). Informed consent was conducted either in-person at the week 0 screening visit or remotely using a UC Health-encrypted Zoom appointment 1 to 6 days prior to the week 0 screening visit. During the informed consent process, regardless of if it is completed remotely or in person, participants reviewed the ICF with a trained staff-member and sign the ICF. If completed remotely, the standard FDA compliant (i.e., Part 11) DocuSign e-signature service that is offered by UCSD campus DocuSign was employed. If participants opted to sign the informed consent remotely, an email with a RedCAP link to study questionnaires (i.e., medical history) and risk assessment for exercise testing will be sent prior to the study visit.

Prior to collection, patients were encouraged to brush their teeth and/or rinse out with drinking water at least 30 min before sample collection to remove food residue and debris. Using the passive drool method, patients were advised to relax and allow saliva to pool underneath the tongue for up to 3 min. Once the patient reached the urge to swallow, they were instructed to tilt their head forward and drool through the funnel into a cryovial tube until the desired volume was reached (~2.5 mL). Patients were advised not to cough or sniff before saliva collection, as this encourages phlegm in the sample, which can interfere with the test. Additionally, patients were advised not to blow or forcibly spit into the cryovial tube during sample collection.

### 2.2 Isolation and enrichment of CNS-derived sEVs

EVs were isolated per manufacturer’s instructions, from 1 mL of sEVs using three different isolation methods: a polymer-based isolation method (ExoQuick-TC), OASIS plus ExoQuick-TC, and Norgen Biotek Saliva Exosome Purification Kit (Reseco et al., 2024) to determine which method would be the most appropriate for the current study. Precipitated pellets generated using the ExoQuick-TC and ExoQuick-TC + Oasis methods were resuspended in 1x PBS before characterization.

Salvia EVs from remaining patient samples were extracted and precipitated using ExoQuick-TC as previously described (Reseco et al., 2024) and enriched against neuronal (L1CAM, NRXN1), astrocyte (GLAST), microglial (TMEM119), and oligodendrocyte (PLP1) sources using magnetic immunocapture and fluorescence-activated cell sorting (FACS). Antibodies utilized in this study included: mouse anti-human CD171 (L1CAM, neural adhesion protein) biotinylated antibody (clone 5G3, eBioscience, San Diego, CA); mouse anti-human Glutamine Aspartate Transporter (GLAST; ACSA-1) biotinylated antibody (Miltenyi Biotec, Inc., Auburn, CA, Catalog # 130–118-984); Purified anti-TMEM119 (Extracellular) Antibody (clone A16075D, Biolegend, San Diego, CA); Polyclonal Goat anti-Human NRXN1 / Neurexin 1 Antibody (aa1524-1537, WB) Catalog #LS-C61771, LS Bio, Newark, CA); Myelin PLP Antibody (PLP1/4259) [Biotin] (Novus Biologicals, LLC, Centennial CO).

Briefly, post sample collection, cellular debris is cleared from saliva by spinning the sample at 2000 x g for 10 min at room temperature. Cleared saliva was stored at three different storage temperatures, room temperature, 4 and - 20 °C for 2 weeks to EV isolation. The EV fraction is precipitated using the ExoQuick-TC™ (EQ, System Biosciences Inc.; Mountain View, CA) according to the manufacturer’s recommendations, but with slight modifications for optimal EV isolation from saliva. Saliva volumes were mixed with ExoQuick-TC™ solution at a ratio of 2:1 and incubated overnight at 4 °C. Next, the mixture is centrifuged at 4 °C at 1500 x g for 1 h. The supernatant is collected, and the samples are centrifuged again at 16,000 x g for 5 min. The resultant pellet is resuspended in 1x phosphate buffer saline (Thermo Fisher Scientific; Catalog # AM9625) with Halt protease and phosphatase inhibitor cocktail EDTA-free (Thermo Fisher Scientific; Catalog # 78443). Samples were stored at −80 °C until cell-specific enrichment of brain-derived EVs.

Enrichment of CNS-specific EVs was performed as previously described (Winston et al., 2019b). To start, 45 μL of 9.1 μm streptavidin magnetic Exo-Flow beads (System Biosciences, Inc.; Catalog # CSFLOWBASICA-1) were incubated with biotinylated antibodies against neuronal (LCAM, NRXN1), astrocyte (GLAST), microglia (TMEM119), and oligodendrocyte (PLP1) sources for 2 h on ice. During the 2-h incubation, the resultant bead-antibody (Ab) complexes were gently flicked every 30 min to ensure mixing. Using a magnetic stand, bead-Ab complexes were washed three times using 1X Bead Wash Buffer (System Biosciences, Inc.; CSFLOWBASICA-1) and then suspended in 300 μL of 1X Bead Wash Buffer and 100 μL of total EVs suspension, rotating overnight at 4 °C. Afterwards, the bead-Ab-EVs (BAE) complexes were washed three times with 1X Bead Wash Buffer and then suspended in 240 μL of Exosome Stain Buffer and 10 μL of Exo-FITC Exosome FACS stain (Systems Biosciences, Inc.; Catalog # CSFLOWBASICA-1) for 2 h on ice. The BAE-FITC complexes were gently flicked every 30 min to ensure mixing. The complexes were subsequently washed three times in 1X Bead Wash Buffer and then suspended in 300 μL of 1X Bead Wash Buffer prior to loading into BD FACS Aria II for sorting. Lastly, BAE-FITC complexes were incubated with 300 μL of Exosome Elution Buffer (System Biosciences, Inc.; Catalog #FLOWBASICA-1) at 25 °C for 1 h to elute EVs from beads. Protein concentrations for eluted EV fractions were determined using a bicinchoninic acid (BCA) Protein Assay kit (Pierce™ BCA Protein Assay Kit, Cat# 23225) and then stored at −80 °C before EV characterization and biomarker quantification.

### 2.3 Characterization and biomarker quantification of sEV cargo and EV-depleted saliva

EV size, purity, and tetraspanin expression were validated using super-resolution microscopy (ONI nanoimager), nanoparticle tracking analysis (NTA), and human-specific enzyme-linked immunosorbent assays (ELISA) as as previously described (Winston et al., 2016; Badhwar et al., 2024; Winston et al., 2019b). CNS-specific sEV fractions were incubated with a 1:1 ratio of MPER (Thermo Fischer) for 10 min prior to biomarker quantification. Concentrations of EV marker Flotilin 1 (Cusabio, American Research Products–Waltham, MA), ATN and inflammatory cytokine biomarkers were quantified using Quanterix Simoa assays [ALZpath p-tau217 Assay, Neurology 2-Plex A (Aβ40, Aβ42)]; human-specific ELISAs [Human TDP43 ELISA Kit (TARDBP), Abcam], and Meso Scale Discovery (MSD) immunoassays [V-PLEX Proinflammatory Panel 1 and S-PLEX Human Tau (pT181)]. The mean value for all determinations of Flotilin 1 in each assay group was set at 1.00, and the relative values for each sample were used to normalize their recovery.

### 2.4 Statistical analysis

All statistics were performed in GraphPad Prism v10.0 (GraphPad Software, San Diego, CA, United States). Data distributions were first examined with the Shapiro–Wilk test to guide the choice of parametric versus non-parametric procedures. Group differences involving two independent samples were evaluated with an unpaired Student’s t-test when normality held or a Mann–Whitney U test when it did not. Experiments with three or more independent groups were analyzed by one-way ANOVA; if the overall *F* value reached significance, pairwise contrasts were resolved with Holm–Šídák post-hoc testing to control the family-wise error rate. Results are expressed as mean ± SEM, and figure legends include exact *p* values and effect sizes (Cohen’s d for two-group tests or η^2^ for ANOVA). Statistical significance was defined as two-tailed *p* < 0.05.

## 3 Results

### 3.1 Protocol comparison and characterization of EVs from human saliva

**Figure 1:**
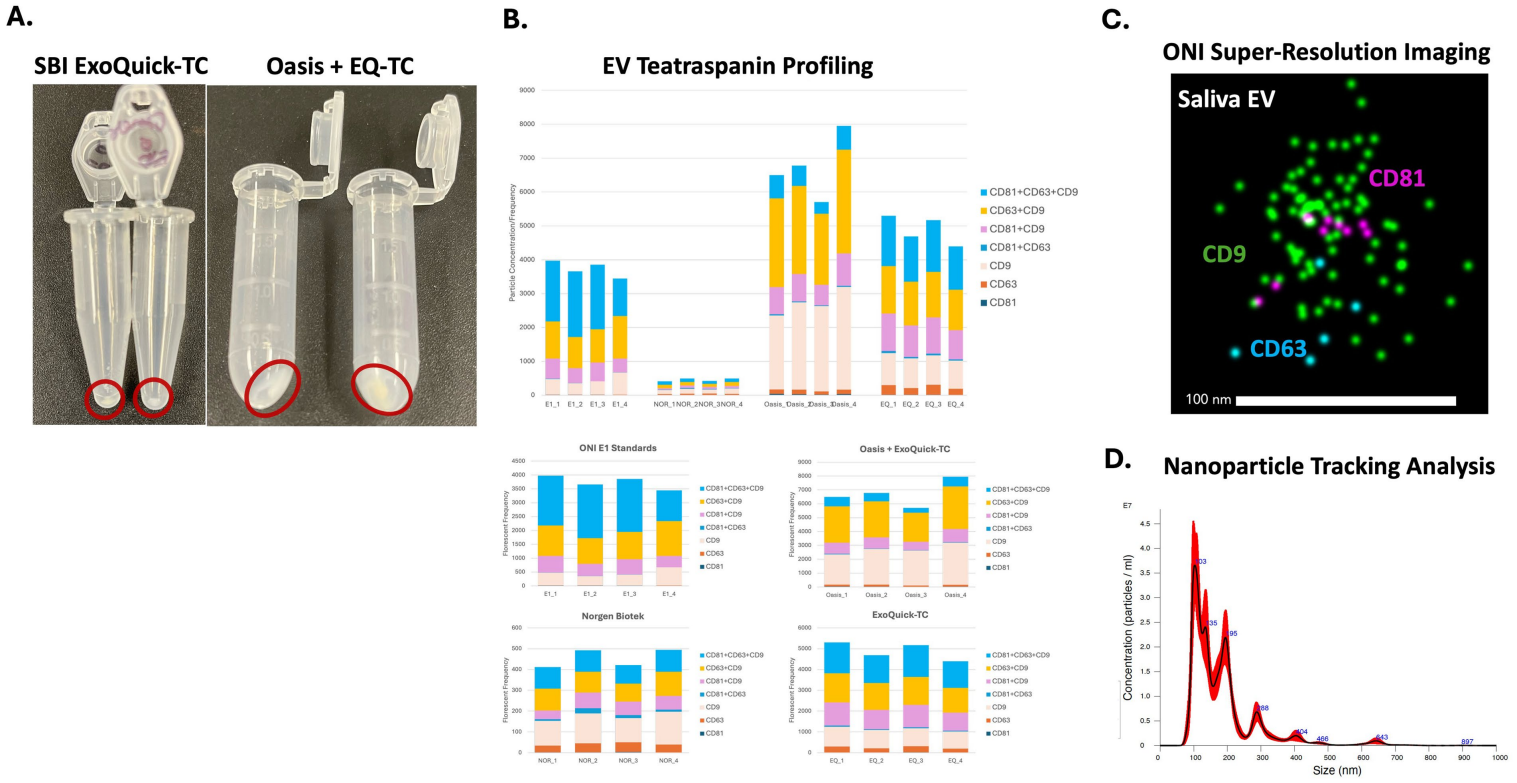
Characterization of EVs from human saliva. Saliva EVs were isolated, per manufacturer’s instructions, using three different isolation methods: ExoQuick-TC, Oasis + ExoQuik-TC, and Norgen Biotek Saliva Exosome Purification Kit. Representative images of precipitated EV pellets generated using ExoQuick-TC and Oasis + ExoQuik-TC isolation methods. No pellet was generated using the Norgen kit **(A)**. Representative histogram of the size distributions of sEVs as measured by NTA **(B)**. Representative images of a single tetraspanin-positive, sEV as measured by ONI Nanoimager at an approximate size of 100 nm. CD9 + (green), CD81 + (purple), and CD63 + (blue), Pan EV (blue) **(C)**. EV tetraspanin profiling of sEVs **(D)**.

In line with previous studies, (Reseco et al., 2024; Bermejo-Pareja et al., 2010), methodological comparison was conducted, with the intended goal of validating previously published methods, using three different sEV isolation techniques: ExoQuick-TC, Oasis + ExoQuik-TC, and Norgen Biotek Saliva Exosome Purification Kit. All procedures were followed manufacturer’s instructions with one slight modification for saliva EVs isolated using ExoQucik-TC (post ExoQuick-TC overnight incubation, resultant preparations were spun at 1,500 g for 1 h at 4 °C followed by high-speed spin at 16,500 g for 5 min). Precipitated EV pellets of varying sizes were generated using ExoQuick-TC and Oasis + ExoQuick-TC isolation methods (Figure 1A), while no pellet, as expected, was generated using the Norgen kit (Figure 1A).

Super-resolution imaging with the ONI Nanoimager was used to validate the presence of canonical EV markers CD63, CD81, and CD9 on sEVs (Figure 1B). Tetraspannin profiling validated particle concentration and EV marker distributions across all three methods (CD81 + CD63 + CD9, CD63 + CD9, CD81 + CD9, CD81 + CD63, CD9, CD63, CD81). We observed that particle concentration and frequency were significantly reduced in sEVs generated using the Norgen kit as compared to ExoQuick-TC and ExoQuick-TC + OASIS methods (Figure 1C). ExoQuick-TC method generated EVs with an equal distribution of CD81 + CD63 + CD9, CD63 + CD9, CD81 + CD9 positive EVs, while the ExoQuick-TC + Oasis method shifted toward a higher proportion of either CD63 + CD9 and CD9 positive EVs. Together, these data suggest that the ExoQuick-TC is a suitable method for isolating EVs from previously collected saliva samples, while coupling this method with the Oasis Pur SAl collection tube can enhance EV isolation from freshly collected saliva. Super-resolution imaging (Figure 1B) and NTA (Figure 1D) further validated the size distribution and concentration of sEVs. Super resolution imaging confirmed the size range of sEVs to between 80–100 nm, while NTA revealed a heterogeneous population of EVs with an average size of 182.8 nm; however, the EV size that appeared the most frequently (mode) was 102.9 nm, which is closer to the expected EV size of 80–100 nm. The larger EV sizes identified by NTA were likely clumped populations of EVs that failed to dissociate in suspension.

**Figure 2:**
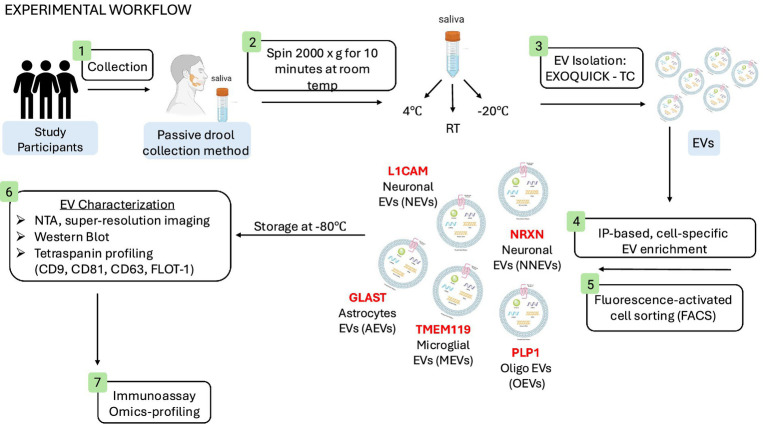
Experimental workflow. Schematic overview for the collection of saliva using the passive drool method from individuals enrolled in the Nathan Shock Healthy Aging Study; the isolation of sEVs using ExoQuick-TC; the enrichment of CNS-derived EVs based on cellular origin; and the downstream characterization of sEVs and molecular cargoes.

**Figure 3:**
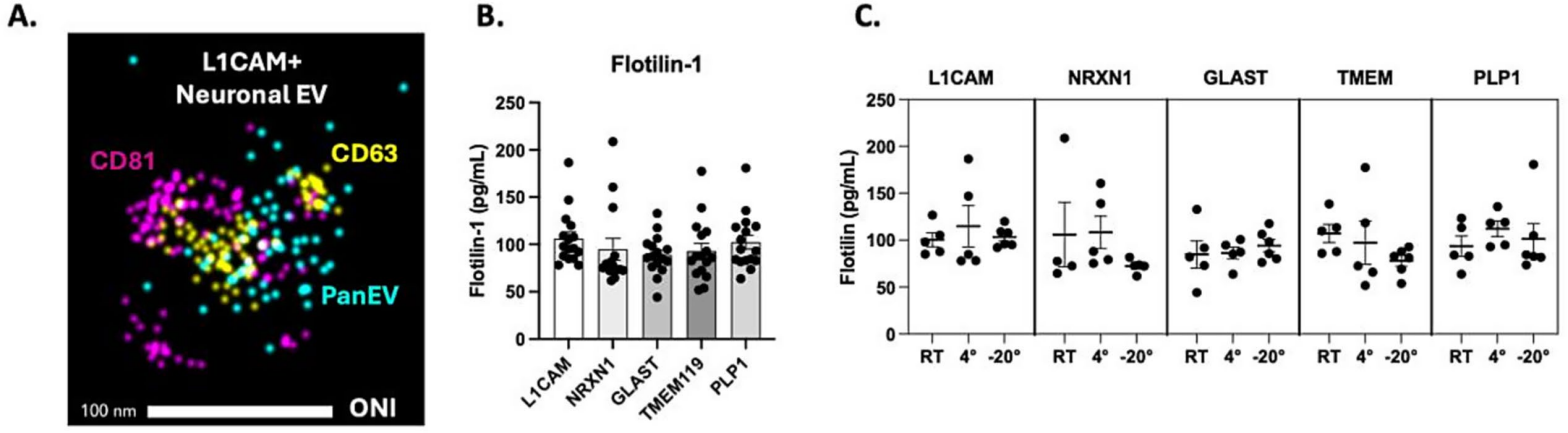
Characterization of CNS-derived extracellular vesicles (EVs) by super-resolution microscopy and ELISA. A representative super-resolution microscopy image of a single neuronal EV (~100 nm) enriched for L1CAM **(A)**. The image confirms the presence of canonical EV tetraspanin markers CD81 (purple), CD63 (yellow), and PanEV (blue). **(B)** ELISA quantification of the EV membrane protein Flotillin 1 in saliva samples. **(C)** Flotillin1 levels were not statistically different between CNS-derived EV subpopulations and remained stable across various storage conditions.

Experimental workflow (Figure 2) reflects saliva sample collections using the passive drool method and subsequently stored at three different temperatures, room temperature (RT), 4 °C, and −20 °C prior to enrichment against CNS-specific sources. Resultant CNS-specific sEV fractions were confirmed by super-resolution imaging (Figure 3A) and ELISA against the EV membrane protein marker, Flotilin (Figure 3B). Super-resolution image of a representative L1CAM-positive sEV (sNEV), labeled with CD81, CD63, and PanEV markers, confirmed successful isolation of sNEVs (Figure 3A). Flotillin-1 expression was quantified across all CNS-derived sEVs, including neuronal (L1CAM, NRXN1), astrocytic (GLAST), microglial (TMEM119), and oligodendrocyte (PLP1) sEVs. sEV expression levels of flotillin-1 were not statistically different between the CNS-specific sEV subpopulations (Figure 3B; L1CAM + sEV, 106.1 ± 7.08 pg./mL; NRXN1 + sEV, 94.87 ± 11.55 pg./mL; GLAST+ sEV, 88.78 ± 5.20 pg./mL; TMEM119 + sEV, 93.10 ± 8.13 pg./mL; PLP1 + sEV; 102.3 ± 7.28 pg./mL). Flotillin-1 expression remained unchanged when CNS-derived EVs were stored under different storage conditions (Figure 3C; Table 1).

**Table 1:** Patient demographics and cognitive battery scores for patients enrolled in Nathan shock healthy aging study included in this study.

Patient demographics (*n* = 15)		
Sex	Males (46.6%)	Females (53.3%)
Age	71.33 ± 1.926	63.25 ± 8.138
Race and ethnicity	Hispanic or Latino: Yes – 16.67%, No – 83.33% Race: White or Caucasian, 100%; Asian, 0%; Black or African American, 0%	Hispanic or Latino: Yes – 50%; No – 50% Race: White or Caucasian, 83.33%; Asian, 16.67%; Black or African American, 0%
Cognition battery NIH toolbox scores		
	Males (46.6%)	Females (53.3%)
FICAT, age corrected	103.83 ± 8.30	84.67 ± 4.38
LSWMT, age corrected	106.67 ± 4.56	107.33 ± 4.76
DCCST, age corrected	119.33 ± 7.56	95.83 ± 6.02
PCPST, age corrected	113.33 ± 8.06	100.67 ± 7.83
PSMT, age corrected	93.67 ± 4.30	111 ± 9.178

Flanker Inhibitory Control and Attention Test (FICAT), List Sorting Working Memory Test (LSWMT), Dimensional Change Card Sort Test, measuring cognitive performance by sorting pictures into different categories (DCCST), Pattern Comparison Processing Speed Test (PCPST), Picture Sequence Memory Test (PSMT).

### 3.2 Impact of cellular origin and storage temperature on sEV concentrations of ATN-related biomarkers

**Figure 4:**
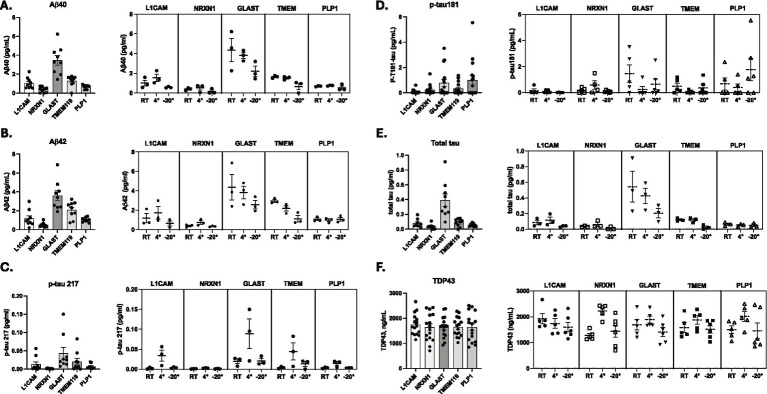
Saliva EV concentrations of ATN biomarkers. Quantification of AD biomarkers (Aβ40 **(A)**, Aβ42 **(B)**, p-tau217 **(C)**, p-tau181 **(D)**, total tau **(E)**, TDP-43 **(F)**) across cellular origin and storage temperature using ultrasensitive immunoassays, SIMOA Quanterix and MSD. Concentrations were compared across CNS-derived sEV fractions (L1CAM (neuronal), NRXN1 (neuronal), GLAST (astrocyte), TMEM119 (microglial), and PLP1 (oligodendrocyte)). Concentrations were then compared across pre-isolation storage temperatures (room temperature, 4 °C, −20 °C) within each fraction to find the optimal storage condition.

Concentrations of Aβ40 (Figure 4A), Aβ42 (Figure 4B), p-tau217 (Figure 4C), p-tau181 (Figure 4D), total tau (Figure 4E), and TDP-43 (Figure 4F) were quantified in CNS-derived sEVs. All ATN biomarkers except p-tau217 were detectable across sEV fractions. Concentrations of Aβ40 (Figure 4A), Aβ42 (Figure 4B), and total tau (Figure 4E) were elevated in EVs from astrocyte (GLAST) sources. Concentrations of p-tau181 (Figure 4D) were also elevated in sEVs from astrocyte sources but were most elevated in sEVs from oligodendrocyte (PLP1) sources. Within the neuronal-derived EV populations, L1CAM-positive sEVs had low concentrations of all ATN biomarkers except for Aβ40, Aβ42, and TDP-43, while TDP-43 was the only highly detectable ATN biomarker in NRXN1-positive EVs (Figure 4F). Aβ40, Aβ42, and TDP-43 were the only ATN biomarkers with elevated concentrations in TMEM119-positive EVs. TDP-43 expression was highly detectable across all CNS-derived sEVs fractions (Figure 4F).

Next, we assessed how pre-isolation storage temperature impacted sEV biomarker detectability. We determined that storage temperature had variable impacts on detecting sEV concentrations across all ATN biomarkers in all CNS-derived sEV fractions. For Aβ40 (Figure 4A), Aβ42 (Figure 4B), and total tau (Figure 4E), storage temperature had the most impact on biomarker concentrations from GLAST sources, in which GLAST-positive EV fractions stored at room temperature had the highest concentrations of Aβ40 (Figure 4A), Aβ42 (Figure 4B), and total tau (Figure 4E) as compared to 4 °C and −20 °C. L1CAM-positive EVs stored at 4 °C had higher concentrations of Aβ40 (Figure 4A), Aβ42 (Figure 4B), and total tau (Figure 4E), as compared to room temperature and −20 °C. PLP1-positive EV fractions stored at −20 °C had the highest concentrations of p-tau181 (Figure 4D), as compared to 4 °C and room temperature. In TMEM19-positive EVs, concentrations of Aβ40 (Figure 4A), Aβ42 (Figure 4B), and total tau (Figure 4E) decreased as storage temperature decreased. In NRXN1-positive EVs, concentrations for each biomarker were similar across all storage temperatures (Figure 4). TDP-43 was the only ATN biomarker with similar concentrations in all sEV fractions across all storage temperatures (Figure 4F).

### 3.3 Impact of cellular origin and storage temperature on sEV concentrations of inflammatory cytokines

Given that inflammation is a critical pathological feature of neurodegeneration, we assessed whether saliva can reliably capture specific inflammatory cytokine concentrations within CNS-derived sEVs while categorizing them by their roles in immune function, pro-inflammatory, anti-inflammatory, and immune-regulatory functions (Dinarello, 2007; Kany et al., 2019; Luzina et al., 2012; Bachmann and Oxenius, 2007).

**Figure 5:**
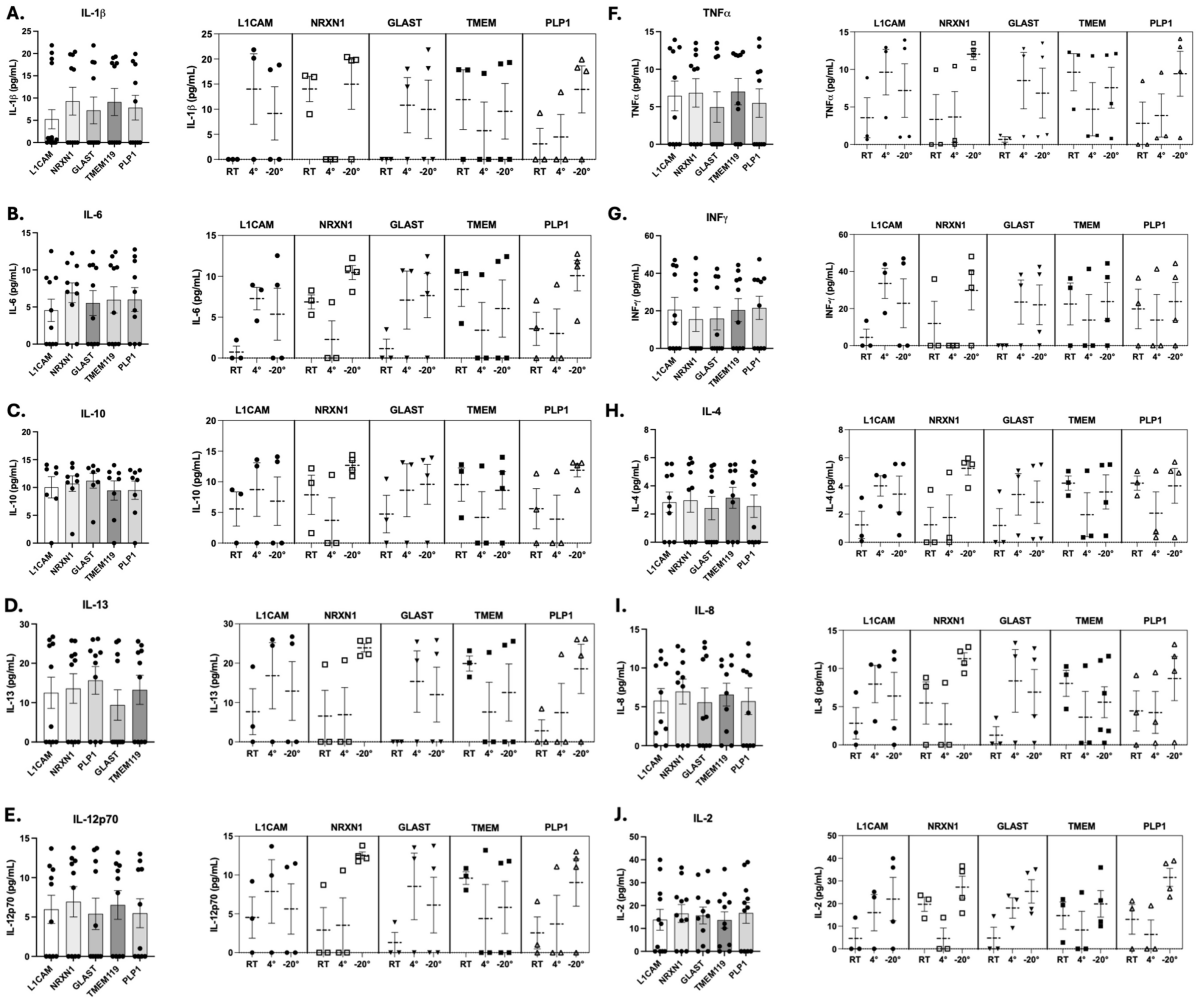
Saliva EV concentrations of inflammatory cytokine biomarkers. Quantification of proinflammatory cytokine biomarkers (IL-1β **(A)**, IL-6 **(B)**, IL-10 **(C)**, IL-13 **(D)**, IL-12p70 **(E)**, TNF-α **(F)**, INF-γ **(G)**, IL-4 **(H)**, IL-8 **(I)**, and IL-2 **(J)**) across cellular origin and storage temperature using MSD V-Plex Proinflammatory Panel 1. Concentrations were compared across CNS-derived sEV fractions (L1CAM, NRXN1, TMEM119, and PLP1). Concentrations were then compared across pre-isolation storage temperatures (room temperature, 4 °C, −20 °C) within each fraction to find the optimal storage condition.

To start, six pro-inflammatory cytokines including: IL-1β (Figure 5A), IL-6 (Figure 5B), IL-12p70 (Figure 5E), TNF-*α* (Figure 5F) IFN-*γ* (Figure 5G), and IL-8 (Figure 5I) were quantified in all CNS-specific sEV fractions. This approach builds on previous research in Parkinson’s disease, which demonstrated saliva’s utility in capturing disease-related inflammatory markers alongside blood and CSF (Chahine et al., 2014). All cytokines were detectable across neuronal (NRXN1, L1CAM), astrocytic (GLAST), microglial (TMEM119), and oligodendrocyte (PLP1) sEV fractions, confirming the feasibility of this platform for cytokine quantification. Specific distribution patterns emerged: IL-1β, IL-8, and IL-12p70 concentrations were most elevated in NRXN1-positive and TMEM119-positive sEVs, while decreased concentrations of IL-1β were observed in L1CAM-positive sEVs. IL-6 concentrations were highest in NRXN1-positive sEVs, and IFN-γ reached its highest levels in PLP1-positive, L1CAM-positive, and TMEM119-positive fractions. In contrast, TNF-α concentrations were highest in NRXN1-positive and TMEM119-positive sEVs but notably decreased in GLAST-positive and PLP1-positive sEVs. While we observed elevated concentrations of these pro-inflammatory markers across various fractions, there were no statistically significant differences between the CNS-derived sEV subpopulations tested in this proof-of-concept validation study (Table 2).

**Table 2:** Quantification of ATN biomarkers and inflammatory cytokines in CNS-derived sEVs and EV-depleted saliva irrespective of storage temperature.

Biomarker	EV-depleted saliva (pg/mL ± S. E. M)	L1CAM (pg/mL ± S. E. M)	NRXN1 (pg/mL ± S. E. M)	GLAST (pg/mL ± S. E. M)	TMEM119 (pg/mL ± S. E. M)	PLP1 (pg/mL ± S. E. M)
Aβ40	0.91 ± 0.10	1.04 ± 0.12	0.35 ± 0.10	3.45 ± 0.50	1.28 ± 0.19	0.66 ± 0.06
Aβ42	0.91 ± 0.12	1.18 ± 0.29	0.48 ± 0.08	3.59 ± 0.52	2.09 ± 0.30	1.03 ± 0.08
p-tau217	0.02 ± 0.01	0.013 ± 0.01	0.0014 ± 0.01	0.04 ± 0.02	0.02 ± 0.01	0.0067 ± 0.01
total tau	0.02 ± 0.007	0.07 ± 0.02	0.04 ± 0.01	0.30 ± 0.08	0.087 ± 0.02	0.054 ± 0.01
p-tau181	2.28 ± 0.46	0.077 ± 0.039	0.23 ± 0.10	0.77 ± 0.28	0.31 ± 0.11	0.99 ± 0.38
TDP-43	8,070 ± 912	1744 ± 106	1,633 ± 139	1,648 ± 106	1,645 ± 95.4	1,643 ± 144
IL-1β	1.43 ± 0.83	5.26 ± 2.16	9.30 ± 3.13	7.23 ± 3.00	9.12 ± 3.04	7.84 ± 2.77
IL-6	0.18 ± 0.02	4.55 ± 1.51	6.93 ± 1.33	5.54 ± 1.68	5.96 ± 1.76	6.01 ± 1.61
IL-10	0.25 ± 0.02	10.0 ± 1.91	10.7 ± 1.39	11.2 ± 1.33	9.46 ± 1.74	9.52 ± 1.65
TNF-α	0.22 ± 0.04	6.45 ± 1.98	6.85 ± 1.90	4.96 ± 2.03	7.02 ± 1.76	5.49 ± 1.90
INF-γ	2.66 ± 0.26	20.6 ± 6.60	15.5 ± 6.46	15.9 ± 6.07	20.4 ± 6.12	21.6 ± 6.16
IL-4	0.06 ± 0.01	2.84 ± 0.72	2.97 ± 0.84	2.42 ± 0.82	3.15 ± 0.74	2.56 ± 0.80
IL-13	0.88 ± 0.09	12.5 ± 3.98	13.6 ± 3.75	9.41 ± 3.87	13.3 ± 3.69	15.7 ± 3.53
IL-12p70	0.24 ± 0.03	5.97 ± 1.75	6.94 ± 1.93	5.40 ± 1.99	6.53 ± 1.82	5.48 ± 1.84
IL-8	1.65 ± 1.10	5.79 ± 1.56	6.97 ± 1.61	5.58 ± 1.85	6.58 ± 1.46	5.72 ± 1.71
IL-2	0.42 ± 0.04	13.8 ± 4.56	16.5 ± 3.89	15.7 ± 3.70	13.7 ± 3.59	16.8 ± 4.49

Anti-inflammatory cytokines, including IL-10 (Figure 5C) and IL-13 (Figure 5D), and immune-regulatory cytokines, IL-2 (Figure 5J) and IL-4 (Figure 5H), were consistently detectable across all CNS-derived sEV fractions. Notably, IL-10 concentrations were highest in astrocyte (GLAST-positive) sEVs and lowest in oligodendrocyte (PLP1-positive) and microglial (TMEM119-positive) sEVs. IL-13 concentrations were highest in PLP1-positive sEVs, while lower concentrations of IL-13 were observed in L1CAM-positive and GLAST-positive sEVs. IL-2 concentrations were the highest in PLP1-positive and NRXN1-positive sEVs, with decreased concentrations in L1CAM-positive and TMEM119-positive fractions (Figure 5J). IL-4 concentrations were the highest in TMEM119-positive sEVs, with lower concentrations observed in PLP1-positive and GLAST-positive sEVs (Figure 5H),

Similarly to ATN biomarkers, we assessed whether pre-isolation storage temperature affected inflammatory cytokine detectability across CNS-derived sEV fractions. Proinflammatory cytokines IL-1β (Figure 5A), IL-6 (Figure 5B), and TNF-*α* (Figure 5F) and were observed to have the greatest concentrations in NRXN1-positive sEVs when stored at −20 °C. IFN-*γ* was most elevated in NRXN1-positive, TMEM119-positive, and PLP1-positive sEV fractions when stored at −20 °C. IL-12p70 (Figure 5E) and IL-8 (Figure 5I) displayed the most elevated concentrations in NRXN1-positive and TMEM119-positive sEVs at −20 °C.

sEV concentrations of anti-inflammatory cytokines, IL-10 (Figure 5C) and IL-13 (Figure 5D), displayed higher concentrations in PLP1-positive and NRXN1-positive EVs stored at −20 °C compared to 4 °C and RT. IL-10 concentrations were highest in PLP1-positive and NRXN1-positive sEVs, with lower concentrations of IL-10 observed in L1CAM-positive sEV fractions when stored at −20 °C. In contrast, IL-13 concentrations were highest in NRXN-positive sEVs, with lower concentrations of IL-13 present in GLAST-positive and L1CAM-positive fractions when stored at −20 °C. Similarly, sEV concentrations of immune-regulatory cytokines, IL-2 (Figure 5J) and IL-4 (Figure 5H), had higher concentrations when stored at −20 °C. IL-2 had the highest concentrations at −20 °C in PLP1-positive, NRXN1-positive, and GLAST-positive fractions, with lower concentrations observed in TMEM119-positive and L1CAM-positive sEVs. IL-4 had the highest concentration at −20 °C in NRXN1-positive sEVs, with the lowest concentrations observed in GLAST-positive sEVs.

### 3.4 Concentrations of ATN biomarkers and inflammatory cytokines in EV-depleted saliva

**Figure 6:**
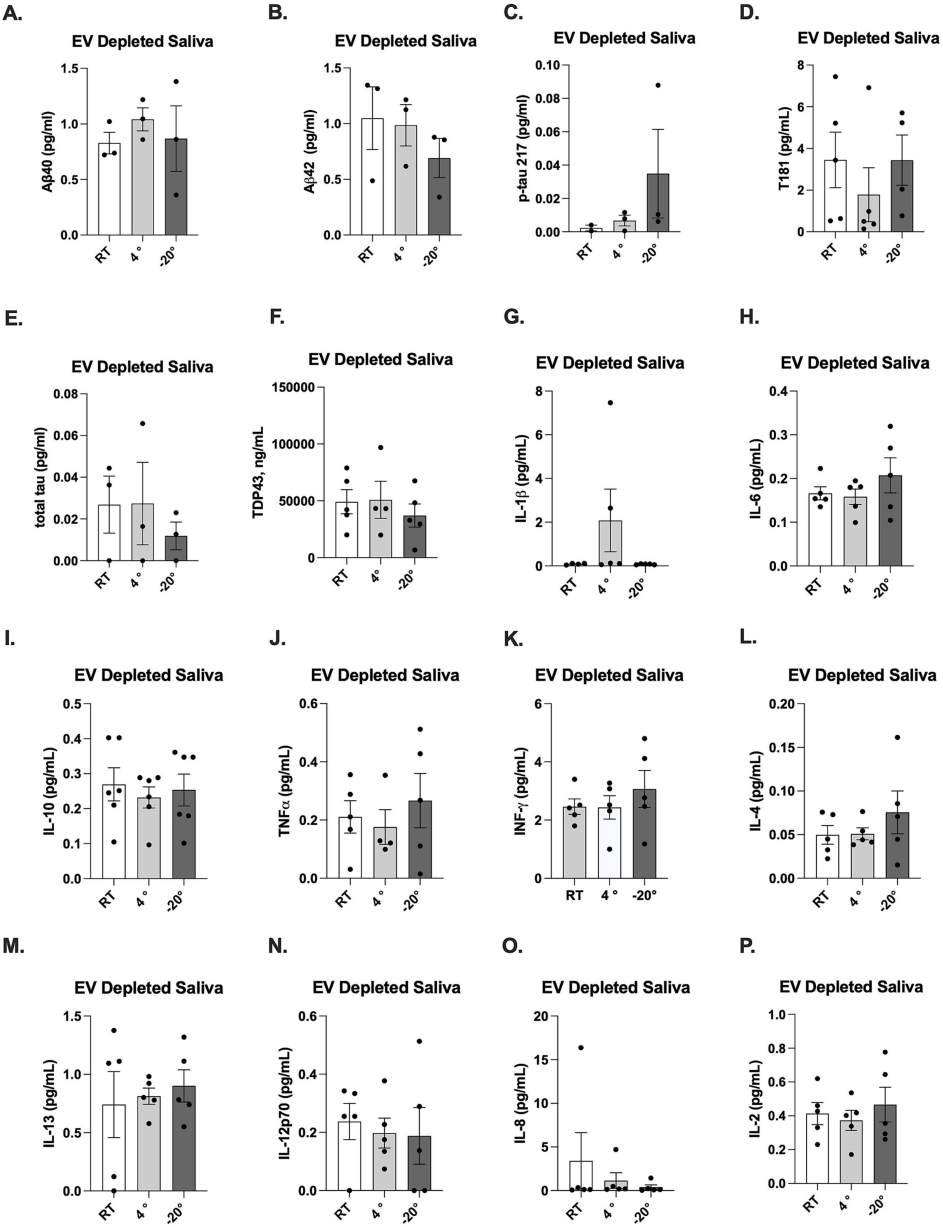
Effect of storage temperature on biomarker concentrations in EV-depleted saliva. Quantification of ATN biomarkers (Aβ40 **(A)**, Aβ42 **(B)**, p-tau217 **(C)**, p-tau181 **(D)**, total tau, TDP-43 **(F)**) and proinflammatory cytokine biomarkers (IL-1β **(G)**, IL-6 **(H)**, IL-10 **(I)**, TNF-α **(J)**, INF-γ **(K)**, IL-4 **(L)**, IL-13 **(M)**, IL-12p70 **(N)**, IL-8 **(O)**, and IL-2 **(P)**) across storage temperature using SIMOA Qunaterix and MSD immunoassays. Concentrations in EV-depleted saliva were compared across pre-isolation storage temperatures (room temperature, 4 °C, −20 °C) to find the optimal storage condition.

ATN biomarkers and inflammatory cytokines were measured in EV-depleted saliva which was defined as the salivary fraction remaining after EV precipitation and the removal. Analyzing this fraction allows for the quantification of biomarkers that are not vesicle-bound, providing a comparative baseline for proteins circulating freely in the saliva. Among the ATN biomarkers measured, Aβ40 (Figure 6A), Aβ42 (Figure 6B), p-tau181 (Figure 6D), and TDP-43 (Figure 6F) were detectable in EV-depleted saliva across all storage temperatures. Concentrations of Aβ40, Aβ42, and total tau were higher in CNS-derived sEV fractions, specifically in GLAST-positive EVs, as compared to EV-depleted saliva (Table 3). Interestingly, higher concentrations of p-tau181 and TDP-43 were detectable in EV-depleted saliva as compared to CNS-derived sEV fractions. In EV-depleted saliva, storage temperature had variable impacts on biomarker concentrations, though long-term storage temperature at −20 °C was optimal for quantification.

**Table 3:** Quantification of ATN biomarkers and inflammatory cytokines in CNS-derived sEVs and EV-depleted saliva.

Biomarker (RT)	EV-depleted saliva (pg/mL ± S. E. M)	L1CAM (pg/mL ± S. E. M)	NRXN1 (pg/mL ± S. E. M)	GLAST (pg/mL ± S. E. M)	TMEM119 (pg/mL ± S. E. M)	PLP1 (pg/mL ± S. E. M)
Aβ40	0.83 ± 0.10	0.99 ± 0.31	0.39 ± 0.08	4.35 ± 1.15	1.64 ± 0.08	0.68 ± 0.03
Aβ42	1.05 ± 0.28	1.18 ± 0.48	0.39 ± 0.08	4.38 ± 1.31	2.94 ± 0.15	1.06 ± 0.14
p-tau217	0.0023 ± 0.01	0.002 ± 0.00	0.0007 ± 0.00	0.02 ± 0.01	0.004 ± 0.00	0.003 ± 0.00
total tau	0.027 ± 0.01	0.08 ± 0.03	0.033 ± 0.005	0.54 ± 0.20	0.12 ± 0.01	0.06 ± 0.01
p-tau181	1.78 ± 1.29	0.15 ± 0.15	0.18 ± 0.08	1.47 ± 0.67	0.50 ± 0.23	0.67 ± 0.46
TDP-43	49,200 ± 10,575	1923 ± 190	1,273 ± 79	1,690 ± 205	1,581 ± 184	1,494 ± 148
IL-1β	2.38 ± 1.54	0.00 ± 0.00	14.03 ± 2.52	0.00 ± 0.00	11.90 ± 5.95	3.08 ± 3.08
IL-6	0.17 ± 0.01	0.74 ± 0.74	6.87 ± 0.86	1.16 ± 1.16	8.40 ± 2.08	3.58 ± 2.03
IL-10	0.27 ± 0.05	5.57 ± 2.79	7.88 ± 3.20	4.74 ± 3.06	9.54 ± 2.73	5.61 ± 3.28
TNF-α	0.19 ± 0.04	2.97 ± 2.97	3.32 ± 3.32	0.00 ± 0.00	9.63 ± 2.47	2.83 ± 2.83
INF-γ	2.47 ± 0.26	4.47 ± 4.47	11.97 ± 11.97	0.00 ± 0.00	22.50 ± 11.34	19.81 ± 10.67
IL-4	0.05 ± 0.01	1.05 ± 1.05	1.24 ± 1.24	1.20 ± 1.20	4.20 ± 0.50	4.20 ± 0.50
IL-13	0.74 ± 0.28	7.67 ± 5.83	6.56 ± 6.56	0.00 ± 0.00	19.91 ± 1.93	2.81 ± 2.81
IL-12p70	0.24 ± 0.06	4.53 ± 2.65	2.90 ± 2.90	1.30 ± 1.30	9.60 ± 0.82	2.54 ± 2.06
IL-8	3.41 ± 2.16	2.83 ± 2.07	5.48 ± 2.76	1.17 ± 1.17	8.05 ± 1.71	4.44 ± 2.63
IL-2	0.41 ± 0.04	4.61 ± 4.61	19.68 ± 3.14	4.79 ± 4.79	14.69 ± 5.98	13.02 ± 6.58
Biomarker (4 °C)	EV-depleted saliva (pg/mL ± S. E. M)	L1CAM (pg/mL ± S. E. M)	NRXN1 (pg/mL ± S. E. M)	GLAST (pg/mL ± S. E. M)	TMEM119 (pg/mL ± S. E. M)	PLP1 (pg/mL ± S. E. M)
Aβ40	1.04 ± 0.11	1.54 ± 0.37	0.50 ± 0.22	3.80 ± 0.30	1.52 ± 0.10	0.75 ± 0.03
Aβ42	0.99 ± 0.19	1.72 ± 0.64	0.73 ± 0.18	3.82 ± 0.65	2.20 ± 0.28	0.97 ± 0.14
p-tau217	0.0067 ± 0.003	0.03 ± 0.01	0.0024 ± 0.00	0.09 ± 0.04	0.04 ± 0.02	0.014 ± 0.005
total tau	0.027 ± 0.02	0.11 ± 0.04	0.06 ± 0.02	0.42 ± 0.10	0.12 ± 0.02	0.05 ± 0.01
p-tau181	3.46 ± 1.33	0.08 ± 0.05	0.58 ± 0.33	0.24 ± 0.24	0.07 ± 0.05	0.39 ± 0.27
TDP-43	50,876 ± 16,310	1742 ± 182	2,226 ± 121	1890 ± 137	1870 ± 162	2011 ± 192
IL-1β	2.07 ± 0.96	14.00 ± 7.02	0.00 ± 0.00	10.80 ± 5.50	5.72 ± 5.72	4.47 ± 4.47
IL-6	0.16 ± 0.01	7.28 ± 1.37	2.28 ± 2.28	7.10 ± 3.55	3.40 ± 3.40	3.03 ± 3.03
IL-10	0.23 ± 0.03	8.72 ± 4.37	3.71 ± 3.71	8.63 ± 4.32	4.20 ± 4.20	3.91 ± 3.91
TNF-α	0.13 ± 0.04	9.63 ± 3.03	3.48 ± 3.48	8.17 ± 4.10	3.94 ± 3.94	3.20 ± 3.20
INF-γ	2.44 ± 0.40	33.58 ± 8.11	0.00 ± 0.00	23.53 ± 11.88	13.81 ± 13.81	13.81 ± 13.81
IL-4	0.05 ± 0.005	4.01 ± 0.73	1.66 ± 1.66	3.24 ± 1.63	1.69 ± 1.69	1.69 ± 1.69
IL-13	0.81 ± 0.07	16.81 ± 8.42	6.91 ± 6.91	15.34 ± 7.80	7.58 ± 7.58	7.43 ± 7.43
IL-12p70	0.20 ± 0.05	7.88 ± 4.09	3.53 ± 3.53	8.54 ± 4.29	4.39 ± 4.39	3.69 ± 3.69
IL-8	1.15 ± 0.60	7.96 ± 2.44	2.71 ± 2.71	8.29 ± 4.18	3.46 ± 3.46	3.16 ± 3.16
IL-2	0.37 ± 0.04	16.01 ± 8.03	4.61 ± 4.61	18.06 ± 4.50	8.31 ± 8.31	6.37 ± 6.37
Biomarker (−20 °C)	EV-depleted saliva (pg/mL ± S. E. M)	L1CAM (pg/mL ± S. E. M)	NRXN1 (pg/mL ± S. E. M)	GLAST (pg/mL ± S. E. M)	TMEM119 (pg/mL ± S. E. M)	PLP1 (pg/mL ± S. E. M)
Aβ40	0.87 ± 0.30	0.58 ± 0.05	0.16 ± 0.15	2.20 ± 0.52	0.67 ± 0.33	0.53 ± 0.19
Aβ42	0.69 ± 0.18	0.63 ± 0.24	0.33 ± 0.03	2.56 ± 0.44	1.12 ± 0.33	1.06 ± 0.19
p-tau217	0.04 ± 0.03	0.004 ± 0.001	0.0009 ± 0.001	0.02 ± 0.01	0.01 ± 0.01	0.0029 ± 0.00
total tau	0.012 ± 0.01	0.04 ± 0.01	0.01 ± 0.01	0.20 ± 0.06	0.02 ± 0.02	0.05 ± 0.02
p-tau181	3.44 ± 1.21	0.032 ± 0.02	0.08 ± 0.03	0.64 ± 0.40	0.37 ± 0.22	1.76 ± 0.86
TDP-43	37,078 ± 1,012	1,598 ± 182	1,439 ± 229	1,412 ± 168	1,511 ± 141	1,459 ± 305
IL-1β	0.07 ± 0.01	9.17 ± 5.30	14.98 ± 5.00	9.98 ± 5.81	9.58 ± 5.53	13.94 ± 4.67
IL-6	0.21 ± 0.03	5.37 ± 3.18	10.46 ± 0.87	7.65 ± 2.71	6.06 ± 3.50	10.09 ± 1.87
IL-10	0.25 ± 0.05	6.84 ± 3.95	12.70 ± 0.78	9.59 ± 3.26	8.61 ± 3.05	11.89 ± 1.08
TNF-α	0.27 ± 0.06	6.68 ± 3.86	12.03 ± 0.72	6.28 ± 3.65	7.37 ± 2.90	9.21 ± 3.21
INF-γ	3.07 ± 0.64	22.88 ± 13.22	29.79 ± 10.50	22.04 ± 10.74	23.79 ± 10.26	23.79 ± 10.26
IL-4	0.08 ± 0.02	3.30 ± 1.38	5.26 ± 0.48	2.73 ± 1.58	3.46 ± 1.31	3.93 ± 1.32
IL-13	0.90 ± 0.14	12.92 ± 7.47	23.90 ± 1.07	12.02 ± 6.97	12.54 ± 7.25	18.55 ± 6.26
IL-12p70	0.19 ± 0.10	5.63 ± 3.25	12.53 ± 0.44	6.13 ± 3.59	5.83 ± 3.37	9.04 ± 3.04
IL-8	0.39 ± 0.18	6.39 ± 3.10	11.28 ± 0.78	6.86 ± 3.00	7.81 ± 2.27	8.60 ± 2.96
IL-2	0.47 ± 0.07	22.00 ± 9.58	27.26 ± 4.90	25.39 ± 5.15	19.86 ± 5.90	31.58 ± 3.99

Similarly, all inflammatory cytokines (Figure 6) were detectable in EV-depleted saliva across all storage temperatures, except for IL-1β (Figure 6G), which was detectable at 4 °C, and IL-8, which was detectable at room temperature. Inflammatory cytokines, including IL-1β, IL-6, IL-10, TNF-α, IFN-*γ*, IL-4, and IL-12p70, also displayed higher concentrations in the CNS-derived sEV fractions compared to EV-depleted saliva (Table 3).

### 3.5 Correlation between saliva concentrations of inflammatory cytokines with cognitive performance

Preliminarily, we assessed whether EV-depleted saliva concentrations of ATN and inflammatory cytokine biomarkers correlated with cognitive function. Participants in the Nathan Shock Healthy Aging study were administered a series of cognitive assessments that measure cognitive, emotional, sensory, and motor functions through the NIH Toolbox. Four of these standardized cognitive tests were administered to all study participants, including the Flanker Inhibitory Control and Attention Test (FICAT), Pattern Comparison Processing Speed Test (PCPST), List Sorting Working Memory Test (LSWMT), and Dimensional Change Card Sort Test (DCCST). The FICAT tests a participant’s visual selective attention and inhibitory control by having the participant focus on a specific stimulus while being distracted (Zelazo et al., 2014). The PCPST measures the participant’s processing speed by asking them whether presented pictures are the same or not (Carlozzi et al., 2015). The LSWMT measures a participant’s working memory by having them sequence visual and auditory stimuli (Tulsky et al., 2014). The DCCST quantifies executive category switching by having participants sort pictures into matching categories, including shape or color (Weintraub et al., 2014).

**Figure 7:**
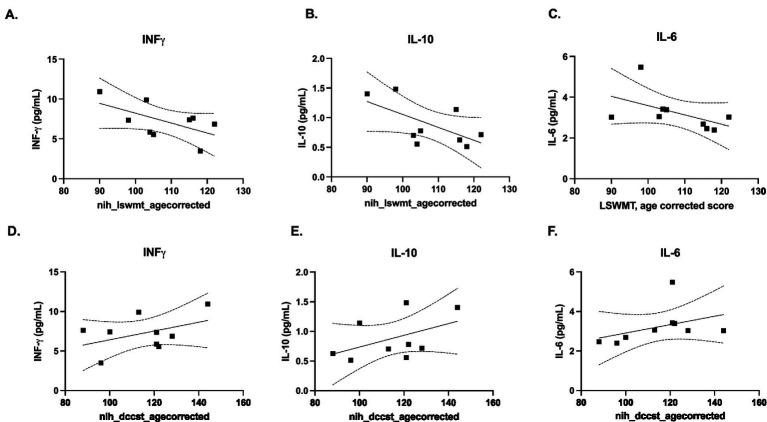
Correlation between saliva concentrations of inflammatory cytokines with cognitive performance. Participants in the Nathan Shock Healthy Aging Study were administered the List Sorting Working Memory Test (LSWMT) (**A-B**), which measures a participant’s working memory, and the Dimensional Change Card Sort Test (DCCST), (**D-F**) which quantifies the executive category switching. These cognitive tests were compared in relation to concentrations of some inflammatory cytokines (INF-γ (**A,D**), IL-10 (**B, E**), and IL-6, (**C, F**)).

We determined there were no correlations between EV-depleted saliva concentrations of ATN biomarkers and cognitive performance; however, we did observe correlations between EV-depleted saliva concentrations of select inflammatory cytokines with cognitive performance for two of the NIH Toolbox tests. We determined that a higher score in the LSWMT correlated with lower concentrations of INF-γ (Figure 7A), IL-10 (Figure 7B), and IL-6 (Figure 7C), demonstrating that having better working memory is associated with having less inflammation. However, a higher score in the DCCST correlated with higher concentrations of INF-γ (Figure 7D), IL-10 (Figure 7E), and IL-6 (Figure 7F), suggesting that minimal inflammation might be associated with better cognitive performance. For INF-γ, IL-10, and IL-6, no correlations were found with the FICAT and PCPST.

## 4 Discussion

Our primary aim for this proof-of-concept technical validation study was to establish and validate an efficient sEV enrichment workflow with the intended purpose of characterizing sEVs as robust reserviors of ATN biomarkers. Guided by Reseco et al. (2024), who reported high efficiency using magnetic bead immunocapture with both Norgen’s Saliva Exosome Purification Kit and ExoQuick-TC ULTRA, we attempted to replicate these protocols in the current study (Reseco et al., 2024). Contrary to their findings, we were unable to isolate a high concentration of sEVs using the Norgen kit; however, the ExoQuick-TC method was successfully validated toward the isolation enrichment of CNS-derived EVs from human saliva. This technical success is a prerequisite for future clinical applications, futhermore supporting Nazir (2024) conclusion that properly isolated saliva biomarkers can track age-related pathology and facilitate early AD diagnosis (Nazir, 2024).

Secondly, extending beyond pour prior work on plasma NEVs and AEVs (Badhwar et al., 2024; Winston et al., 2019a; Winston et al., 2022; Winston et al., 2016), we examined how cellular origin influenced ATN biomarker concentrations across five CNS-derived sEV fractions. While Aβ40, Aβ42, and TDP-43 were consistently detected across all CNS-specific sEV fractions, biomarker yield varied significantly by cell type. Notably, GLAST-positive (astrocytic) and PLP1-positive (oligodendrocyte) sEVs exhibited the highest concentrations of Aβ40, Aβ42, p-tau181, and TDP-43. The prominence of ATN concentrations within GLAST-positive sEVs aligns with astrocytes’ presence are the most abundant glial population, capable of transporting pathological cargo and exhibiting a robust capacity for EV release (Liu et al., 2022), as well as our previous findings of elevated TDP-43 in plasma AEVs as compared to plasma NEVs and MEVs (Winston et al., 2022). Similarly, the rich cargo of PLP1-positive EVs highlights oligodendrocytes as an untapped reservoir of AD biomarkers. Oligodendrocytes are directly impaired in AD (Tylek and Basta-Kaim, 2025) and possess high expression levels of amyloidogenic pathway genes (Sasmita et al., 2024; Ishii et al., 2024), making their EVs likely carriers of relevant myelin-associated antigens (Casella et al., 2020) and TDP-43 (Prater et al., 2023). TMEM-119 positive (microglial) sEVs contained detectable levels of Aβ40, Aβ42, and TDP-43 but lacked the broader biomarker profile that may reflect potential diagnostic utility, as what was observed in the GLAST-positive and PLP1-positive EV subpopulations. Comparing gold-standard L1CAM-positive and newly investigated NRXN1-positive (neuronal) sEVs, concentrations of Aβ40, Aβ42, and TDP-43 were higher in L1CAM-positive versus NRXN1-positive EVs, suggesting L1CAM-positive EVs are the more robust EV neuronal markers for detecting ATN biomarkers in saliva. Although our samples were drawn from cognitively normal individuals, astrocytes and oligodendrocytes appear to be the most robust baseline reservoirs for ATN biomarkers. While neuronal and microglial fractions may require further optimization for salivary analysis, the ubiquitous detection of Aβ40, Aβ42, and TDP-43 across all cellular origins validates their potential as stable ATN biomarkers. Interestingly, p-tau217 was undetectable while detectable levels of p-tau181 concentrations also varied significantly across the all CNS-derived sEV fractions.

The lack of p-tau217 detection is particularly noteworthy, as this analyte has emerged as one of the most specific and sensitive biomarkers of AD pathology (Rissman et al., 2023), demonstrating strong associations with Aβ tau aggregation, and disease progression across multiple biofluids. Compared to other phosphorylated tau isoforms, p-tau217 has been reported to show superior discriminatory power between AD and other neurodegenerative conditions (Palmqvist et al., 2020). Consequently, the absence of detectable p-tau217 in sEVs warrants consideration of several potential explanations. These include (1) the fact that our cohort consisted of cognitively unimpaired individuals where p-tau217 levels are expected to be baseline or negligible; (2) unique degradation patterns in saliva during sample processing or storage may make p-tau217 particularly vulnerable to degradation (Ng et al., 2025); (3) or potential assay sensitivity limits for this specific protein in a salivary matrix (Fu et al., 2010).

In this study, p-tau217 was quantified using a multiplex immunoassay on the Simoa Quanterix platform, which operates with strictly defined lower limits of quantification. Prior comparisons have demonstrated that low-abundance analytes in human samples frequently fall below these detection thresholds (Fu et al., 2010). It is therefore possible that p-tau217 concentrations within sEVs were present but fell below the assay’s lower limit of quantification. Future studies will focus on optimizing this platform for the detection of p-tau217 and other critical tau isoforms, such as p-tau231, p-tau205 and MTBR-tau, in both whole saliva and CNS-specific sEVs. In addition, measuring key ATN-related biomarkers on alternative platforms will be considered as a future direction. Recently, the highly sensitive Lumipulse assay was used to reliably quantify phosphorylated tau (p-tau) proteins in saliva and detected comparable levels to those found in cerebrospinal fluid (CSF; Marksteiner et al., 2022). This suggests that ultra-sensitive assays may be necessary for the reliable detection of p-tau217, particularly in cognitively unimpaired individuals who inherently possess low p-tau217 concentrations. However, it is important to note that Marksteiner et al. (2022) measured p-tau biomarkers in undiluted saliva. Consequently, the sEV isolation process used in our study may have further reduced or interfered with the detectability of of p-tau isoforms, including p-tau217, below the limit of detection, potentially explaining the absence of p-tau217 in our enriched samples. In summary, understanding the detectable ranges in saliva across multiple platforms is a viable next step and future direction for our ongoing work.

Our ongoing work, including the findings presented in this study, also aims to assess the feasibility of detecting neuroinflammatory processes in saliva. Neuroinflammation is a critical pathological hallmark and a central mediator of AD progression. Grouping these cytokines by immune function provided a critical framework for interpreting their roles as mediators of AD progression (Mrak and Griffin, 2005; Fillit et al., 1991). Virtually all targeted cytokine biomarkers were reliably detected across all CNS-derived sEV fractions. Quantifying these biomarkers alongside ATN biomarkers confirms the potential of sEVs as a non-invasive medium for monitoring the neuroinflammatory mechanisms underlying AD. However, pre-analytical variables remain a critical factor for accurate interpretation. We observed that storage temperature significantly influences biomarker detectability, with −20 °C emerging as the superior condition for preserving EV cargo integrity and biomarker sensitivity. Consequently, establishing standardized protocols is essential to ensure reproducibility and maximize future studies investigating the clinical utility of saliva biomarkers in AD research.

In addition to profiling CNS-derived sEVs, we quantified ATN and inflammatory biomarkers in EV-depleted saliva to assess their correlation with measures of cognitive function. Similarly, Aβ40, Aβ42, p-tau181, TDP-43, and all inflammatory cytokines were detectable in EV-depleted saliva. Among the ATN biomarkers, Aβ40, Aβ42, and total tau were most elevated in GLAST-positive sEVs, as compared to EV-depleted saliva. Interestingly, all inflammatory cytokines were elevated in all CNS-derived sEV fractions, as compared to EV-depleted saliva. These results suggest that although ATN biomarkers and inflammatory cytokines were detectable in EV-depleted saliva, measuring these biomarkers in EVs may enhance their sensitivity and potential clinical utility as diagnostic biomarkers for AD. In contrast, we observed p-tau181 and TDP-43 as the only biomarkers that displayed significantly elevated concentrations in EV-depleted saliva as compared to all CNS-derived sEV fractions. Thus, not all biomarkers behave similarly, specialized protocols may be required to achieve the highest sensitivities for certain biomarkers.

Due to limited sample starting volume (1 mL), our assessment of cognitive correlations was restricted to the EV-depleted fraction. While future studies aim to analyze sEVs and native, unmanipulated, saliva, this initial analysis yielded distinct preliminary findings. Higher scores on the LSWMT were associated with lower concentrations of IFN-*γ*, IL-10, and IL-6. This aligns with previous findings that working memory, which the LSWMT evaluates, is often negatively impacted by inflammation (Mac Giollabhui et al., 2024). Conversely, higher scores in the DCCST, a measurement of executive control, correlated with higher concentrations of these cytokines. These paradoxical findings in a small cohort of cognitively unimpaired individuals suggest that mild inflammation does not universally reduce cognitive function across all domains. This aligns with Adelantado-Renau et al. (2019), who noted that executive control tasks may be less sensitive to inflammatory markers than other cognitive measures (Adelantado-Renau et al., 2019). In contrast, ATN biomarkers showed no significant correlations with cognitive performance. This is likely because ATN markers represent long-term neurodegenerative changes accumulating over prolonged periods, whereas salivary cytokines reflect more immediate, dynamic inflammatory states (Szabo et al., 2020). Again, the lack of correlation may be attributed to our cohort being cognitively unimpaired and the generally lower detectability of ATN biomarkers in saliva compared to the robust presence of pro-inflammatory markers. Nonetheless, these correlations we observed are exploratory and should be interpreted as hypothesis-generating, as they are limited to EV-depleted saliva and thus cannot be extended to CNS-derived sEVs. Further analyses of CNS-derived sEVs and native saliva are needed to assess whether similar relationships exist between scores of NIH Toolbox tests and inflammatory markers derived from the CNS. Moving forward, investigating saliva as a source for inflammatory signature assessment remains a priority, given its relevance to numerous neurodegenerative conditions.

The lack of correlation between ATN biomarkers and cognitive performance in our cohort stands in contrast to several, albeit variable, prior studies. Recent reports have demonstrated increased levels of Aβ42 in saliva (Lee et al., 2017; Sabbagh et al., 2018) while others report a decrease in saliva levels in patients with AD (McNicholas et al., 2022). One study observed elevated levels of saliva Aβ42 only during the early stages of AD (Bermejo-Pareja et al., 2010), suggesting changes in salivary Aβ42 levels may only serve as potential biomarkers to predict conversion of MCI to AD while another successfully detected increased p-tau and total tau levels in the saliva of severe AD patients (Shi et al., 2011). More recently, Carro et al. (2017) found that reduced salivary lactoferrin (Lf) levels correlate with the progressive decline of MCI and AD (Carro et al., 2017). These findings suggest salivary Lf could serve as an early, non-invasive biomarker, potentially reflecting immune alterations associated with the disease. In various follow-up studies (Reseco et al., 2024; González-Sánchez et al., 2020; Antequera et al., 2024), the same group determined that saliva Lf demonstrated good diagnostic performance to detect cerebral Aβ load using amyloid PET neuroimaging in prodromal AD as compared to healthy controls, while saliva Lf levels are unchanged in patients with frontotemporal dementia (González-Sánchez et al., 2020). They also determined that saliva Lf related to middle temporal cortical thickening, increased FDG uptake in the posterior cingulate cortex, and poorer memory individuals with the highest Aβ burden (McNicholas et al., 2022). Conversely, Gleerup et al. (2021) concluded that Lf in both CSF and saliva was not a reliable diagnostic biomarker in a mixed memory clinic population, finding no significant relationship between Lf and key markers like Aβ42 and tau (Gleerup et al., 2021). Although Lf was not examined in the current study, investigating Lf concentrations specifically within CNS-derived sEVs may help reconcile these discordant findings by isolating brain-specific signals from total salivary content.

Despite the findings of this study, multiple limitations should be acknowledged. First, the restricted sample size constrains statistical power and limits the applicability of our results to the broader population. Additionally, while we examined pre-isolation storage temperature, other pre-analytical variables such as pre-collection precautions (e.g., food contaminants and smoking) and post-collection variables, including centrifugation time and speed, saliva starting volume, freeze–thaw cycles, and post-isolation storage stability require further exploration (Ng et al., 2025). Although we stored isolated sEVs at −80 °C to maintain stability, future studies should systematically optimize these processing conditions to improve test–retest reliability.

Additionally, the NIH Toolbox tests have demonstrated variable construct validity despite their use. Prior work has shown that NIH Toolbox tests may exhibit poor construct validity for attention, processing speed, episodic memory, motor dexterity, and working memory domains (Ott et al., 2022). These cognitive tests may not isolate the intended cognitive constructs well nor show strong agreement with gold-standard cognitive assessments. The Montreal Cognitive Assessment (MoCA) and Mini-Mental State Examination (MMSE; Arevalo-Rodriguez et al., 2015; Pinto et al., 2019) are the most widely used and clinically accepted cognitive screening tools in medical practice and should be considered for future studies.

Biological confounders also warrant consideration. Since saliva composition changes with age (Nazir, 2024) thus distinguishing between normative age-related variations and disease related pathology remains a challenge and requires further investigation. Saliva composition may vary by sex and metabolic health status, warranting the future investigation. Our cohort lacked racial and ethnic diversity, consisting almost entirely of non-Hispanic White participants. Expanding representation is critical, as biomarker expression may vary significantly across diverse populations (Kjærgaard et al., 2025). While EV-depleted saliva demonstrated some utility in the quantification of ATN and inflammatory cytokine biomarkers in the current study, our findings suggest biomarker sensitivity may be enhanced when these measures are quantified within specific CNS-derived sEV fractions. Finally, although the participant cohort in this study was primarily cognitively unimpaired, future research must compare CNS-derived sEVs in unmanipulated (native) saliva from healthy controls against individuals across the full AD spectrum. Collectively, such studies should account for critical biological variables including age, sex, race and ethnicity, APOE4 carrier status, and metabolic status (i.e., hypertension, diabetes, BMI) to accurately evaluate AD-associated changes and push the field toward non-invasive diagnostic utility.

In summary, this study provides a critical proof-of-concept for the pre-analytical characterization of CNS-derived sEVs from saliva. Our research has demonstrated that ATN and pro-inflammatory biomarkers are consistently detectable in sEVs across a wide adult age range, as evidenced by participants in the Nathan Shock Healthy Aging Study who ranged from 29 to 80 years old. By quantifying ATN and inflammatory cytokines within CNS-specific sEVs, future research can further investigate their potential to serve as a source for early detection, intervention, and improved diagnosis of AD and other related dementias (ADRD). To enhance clinical relevance, future studies should also incorporate established cognitive screening tools, such as the MoCA and MMSE, to more comprehensively characterize the cognitive status of patients. Furthermore, the ability to use saliva-derived analytes to correlate directly with amyloid pathology in the brain will significantly push the field forward, providing a non-invasive methodology to validate CNS health against gold-standard neuroimaging. Although these initial findings in the current study do not yet establish full diagnostic or prognostic utility, they strongly support the feasibility of utilizing sEVs as a non-invasive biofluid with an abundant source of biomarkers. With continued large-scale studies and expanded comparisons between saliva and blood-based biomarkers, saliva remains a highly promising and accessible alternative biofluid for future AD research and clinical diagnostics.

## Data Availability

The raw data supporting the conclusions of this article will be made available by the authors, without undue reservation.
